# Neighborhood socioeconomic status, Medicaid coverage and medical management of myocardial infarction: Atherosclerosis risk in communities (ARIC) community surveillance

**DOI:** 10.1186/1471-2458-10-632

**Published:** 2010-10-21

**Authors:** Randi E Foraker, Kathryn M Rose, Eric A Whitsel, Chirayath M Suchindran, Joy L Wood, Wayne D Rosamond

**Affiliations:** 1 Division of Epidemiology, The Ohio State University, 320 West 10th Avenue, Columbus, OH 43210, USA; 2 Department of Epidemiology, University of North Carolina at Chapel Hill, 135 Dauer Drive, Chapel Hill, 27599, USA; 3 Department of Biostatistics, University of North Carolina at Chapel Hill, 135 Dauer Drive, Chapel Hill, 27599, USA

## Abstract

**Background:**

Pharmacologic treatments are efficacious in reducing post-myocardial infarction (MI) morbidity and mortality. The potential influence of socioeconomic factors on the receipt of pharmacologic therapy has not been systematically examined, even though healthcare utilization likely influences morbidity and mortality post-MI. This study aims to investigate the association between socioeconomic factors and receipt of evidence-based treatments post-MI in a community surveillance setting.

**Methods:**

We evaluated the association of census tract-level neighborhood household income (nINC) and Medicaid coverage with pharmacologic treatments (aspirin, beta [β]-blockers and angiotensin converting enzyme [ACE] inhibitors; optimal therapy, defined as receipt of two or more treatments) received during hospitalization or at discharge among 9,608 MI events in the ARIC community surveillance study (1993-2002). Prevalence ratios (PR, 95% CI), adjusted for the clustering of hospitalized MI events within census tracts and within patients, were estimated using Poisson regression.

**Results:**

Seventy-eight percent of patients received optimal therapy. Low nINC was associated with a lower likelihood of receiving β-blockers (0.93, 0.87-0.98) and a higher likelihood of receiving ACE inhibitors (1.13, 1.04-1.22), compared to high nINC. Patients with Medicaid coverage were less likely to receive aspirin (0.92, 0.87-0.98), compared to patients without Medicaid coverage. These findings were independent of other key covariates.

**Conclusions:**

nINC and Medicaid coverage may be two of several socioeconomic factors influencing the complexities of medical care practice patterns.

## Background

Pharmacologic treatments are efficacious in reducing post-myocardial infarction (MI) morbidity and mortality[1-4]. The prescription of evidence-based treatments such as aspirin, beta-adrenergic blocking agents (β-blockers) and angiotensin-converting enzyme (ACE) inhibitors is recommended by the American College of Cardiology (ACC)/American Heart Association (AHA)[5] and is currently monitored for improving hospital quality of care for all patients following MI[6]. Overall, the prescription of these effective pharmacologic agents has increased over time among such patients[3,7].

Previous studies have shown that receipt of evidence-based pharmacologic treatments among MI patients differ by race, gender, age, health insurance, and hospital type[8-19]. The potential influence of socioeconomic factors on the receipt of pharmacologic therapy has not been examined via surveillance of hospitalizations for MI in the United States (U.S.), even though healthcare utilization likely influences morbidity and mortality post-MI. Hospital data in the U.S. do not generally include individual measures of socioeconomic status (SES), such as income, education or occupation.

Several investigators have used insurance status as a proxy for individual SES[20-22], and although the validity of this approach is not known, Medicaid coverage, with the exception of limited medical conditions, is only provided to patients below the federal poverty level[23]. The majority of Medicaid beneficiaries have incomes below the poverty line[24], thus, in the absence of other SES information, Medicaid coverage is a reasonable surrogate for low SES, and may be related to the receipt of evidence-based therapies following a MI. For example, acute coronary syndrome patients with Medicaid coverage were less likely to receive guideline-recommended medications and invasive cardiac procedures compared to patients of similar age with health maintenance organization or private insurance coverage[25].

While some researchers treat area-level SES as a substitute for individual SES, evidence suggests that social and environmental contexts play independent roles in health outcomes[26-29] and care[30,31]. The separate influence of area-based SES on health and receipt of evidence-based therapies following a MI could be due to access to primary care and neighborhood clinics, feelings of trust or distrust of medicine among community members, and the quality of medical care provided to the patient by their local hospital.

We examined neighborhood SES as a potential barrier to receipt of evidence-based medical therapy post-MI (receipt of aspirin, β-blockers, ACE inhibitors and optimal therapy) and investigated whether Medicaid coverage is also associated with medical management. We hypothesized that, independent of other key covariates, patients from low SES areas would receive evidence-based treatments less often than patients living in high SES neighborhoods, as would patients with Medicaid coverage compared to patients without Medicaid coverage.

## Methods

We evaluated the association of neighborhood census tract median household income (nINC) with pharmacologic treatments received during hospitalization or at discharge among validated, definite or probable MI patients in a study ancillary to the Atherosclerosis Risk in Communities (ARIC) Community Surveillance Study: *Neighborhood Burden of Coronary Heart Disease (CHD) in Communities (1993-2002)*.

### Study Population

The ARIC study's community-based surveillance of CHD has been ongoing since 1987 and its methods and a thorough description of the study population are detailed elsewhere[32,33]. ARIC community surveillance encompasses the same communities in which ARIC cohort study participants reside. However, it does not include in-person visits, follow-up or regular contact with ARIC cohort participants. In contrast, hospital discharges occurring each calendar year in ARIC study areas are retrospectively reviewed to ascertain CHD-related events. Identified events are classified as definite, probable, suspect, no MI or unclassifiable using information on presenting symptoms, medical history, and pertinent laboratory values abstracted from medical records[33].

Hospitalized MI cases (n = 10,461) included those from the four U.S. ARIC study communities among persons aged 35-74: Washington County, Maryland (MD); Northwest suburbs of Minneapolis, Minnesota (MN); Jackson, Mississippi (MS) and Forsyth County, North Carolina (NC). Patients not of white or black race (n = 135), as well as black patients from MN or MD (n = 145) were excluded because of an inability to make inferences to these groups due to small numbers when divided among exposure categories. Seven patients dying within six hours of admission were also excluded because they were ineligible for treatment. An additional 566 patients were excluded due to missing information on neighborhood SES. The remaining hospitalized MI cases (n = 9,608) were weighted based on ARIC surveillance probability sampling of selected International Classification of Diseases codes[33], resulting in a final weighted sample size of 14,152 cases, which represents the estimated eligible population of cases that would have been studied had probability sampling not been employed.

### Study Exposures

Address data abstracted from the medical record beginning in 1993 were sent to a commercial vendor for geocoding. High geocoding accuracy[34] resulted in 93% exact address matches and 2% additional addresses geocoded to the level of the census tract. The number of census tracts in the ARIC study communities at the time of the 2000 U.S. Census ranged from 31 in Washington County, MD to 75 in Forsyth County, NC, and the number of persons per census tract ranged from 1,492 in Jackson, MS to 1,891 in Washington County, MD (Additional file 1, Table S1). Geocoded cases were linked with year 2000 U.S. Census socioeconomic data for each of the 204 census tracts in order to assign nINC to each MI case[35]. In a concurrent project which utilized ancillary study data investigating rates of MI across the ARIC study communities, we found similar results regardless of whether individual neighborhood-level SES variables or a more complex SES index measure was used, as well as whether overall, community- or race-specific cutpoints were used[35,36]. We grouped nINC into tertiles based on nINC across all four study communities [low (<$33,533), medium ($33,533-50,031) and high (≥$50,032)]. Patients' Medicaid status was abstracted as indicated from the medical record.

### Study Outcomes

Pharmacologic therapy with known efficacy in the context of MI treatment (aspirin, β-blockers, ACE inhibitors, and their combination) was abstracted from the medical record as "given during the hospitalization or at discharge". Optimal therapy was defined as receiving agents in two or more of the three medication classes. AHA/ACC guidelines published at the time these data were collected recommended dietary therapy, physical activity and weight management before prescribing lipid-lowering medication[5]. These non-pharmacologic recommendations and receipt of lipid-lowering medication were not ascertained by ARIC during this period and are therefore not reported.

### Additional Covariates

Covariates included race (black or white); gender; age (<65 years vs. ≥65 years); study community; year of MI (1993-1998 vs. 1999-2003); and hospital type (teaching vs. non-teaching). In addition, the following medical history variables were abstracted from the medical record: current or past history of hypertension, diabetes or heart failure; and presence of cardiac pain.

### Statistical Analyses

Prevalence ratios and 95% confidence intervals (PR, 95% CI) for receipt of pharmacologic therapy post-MI were estimated using weighted Poisson regression to account for potential selection bias introduced by the probability sampling of discharge codes. Generalized estimation equations were used (PROC GENMOD, SAS Institute, Cary, NC) to account for the clustering of MI events within census tracts and within patients[37,38]. The variance of the PR estimates was based on the unweighted number of cases using the generalized estimating equation analysis strategy.

Therapies were examined individually and optimal treatment, as previously defined, was also investigated. Model 1 included nINC, Medicaid status, race, gender, age, study community and year of MI, while Model 2 was comprised of factors in Model 1 plus hospital type (teaching vs. non-teaching), current or past history of hypertension, diabetes or heart failure, and presence of cardiac pain. Effect modification of the nINC/Medicaid - MI therapy relationship (p < 0.05) was examined for race, gender, age, study community and year of MI.

### Ethical Considerations

Institutional Review Board (IRB) approvals were obtained by each participating ARIC study center and the coordinating center. Data for this study were abstracted from medical records and strict data confidentiality was maintained.

## Results

The study population was 34% female, 23% black, and 42% were aged 65 or older. The mean nINC for the study population was approximately $42,000 and 10% were Medicaid recipients. In these data, 7.3% of patients had more than one MI event (range: 2-8). Overall, the proportion of patients receiving selected medications was: 88% for aspirin, 70% for β-blockers and 49% for ACE inhibitors (Table 1). More than three-fourths of MI patients received optimal treatment, defined as receiving agents in two or more of the medication classes. The most prevalent combination of the two medication classes among those treated with optimal therapy were aspirin and β-blockers (83%), followed by aspirin and ACE inhibitors (57%) and ACE inhibitors and β-blockers (46%). Among those treated with optimal therapy, 44% received all three medications.

**Table 1:** Characteristics (%) of the Study Population^a ^by nINC and Medicaid Status: ARIC Community Surveillance (1993-2002)

Characteristic	Overall	Median Household Income (nINC)			Medicaid Status	
		Low	Medium	High	Yes	No
	n = 14,152	n = 4,439	n = 5,556	n = 4,157	n = 1,381	n = 12,771
nINC, mean (U.S. dollars)	$42,059	$23,629	$42,474	$61,189	$29,059	$43,465
Female	34.0	41.4	31.6	29.4	62.2	31.0
Black	23.2	57.2	10.6	3.9	57.8	19.5
Study Community						
Washington Co., MD	16.9	23.6	60.8	15.6	90.4	9.6
Minneapolis, MN	19.5	2.0	33.3	64.7	96.6	3.4
Jackson, MS	24.3	65.5	18.8	15.7	81.1	18.9
Forsyth Co., NC	39.3	28.2	45.7	26.1	92.7	7.3
Age ≥ 65 yr	41.5	38.1	44.0	41.7	61.3	58.2
Health Insurance Status						
Medicaid	9.8	21.5	5.4	3.0	-	-
Cardiac Pain	87.5	87.3	86.2	89.5	81.4	88.2
Hospital Type, Teaching	36.9	34.1	29.6	49.6	37.2	36.8
Diabetes	32.4	39.8	31.1	26.4	52.5	30.3
Hypertension	63.7	74.7	60.2	56.7	82.1	61.7
Heart Failure	30.4	36.2	29.6	25.3	47.5	28.5
Medications						
Optimal Therapy	78.0	75.6	78.7	79.6	71.0	78.8
Aspirin	87.5	83.2	89.1	90.0	75.5	88.8
β-blockers	69.5	62.2	71.5	74.7	59.3	70.6
ACE Inhibitors	49.5	56.3	46.8	45.9	59.3	48.5

^a^Weighted to account for sampling strategy

Patient sociodemographic and medical history characteristics, overall and stratified by nINC and Medicaid status, are shown in Table 1. In general, patients from low nINC areas and patients with Medicaid coverage had a higher prevalence of comorbidities and a lower level of treatment compared to patients from high nINC areas and those without Medicaid coverage, respectively.

**Figure 1:**
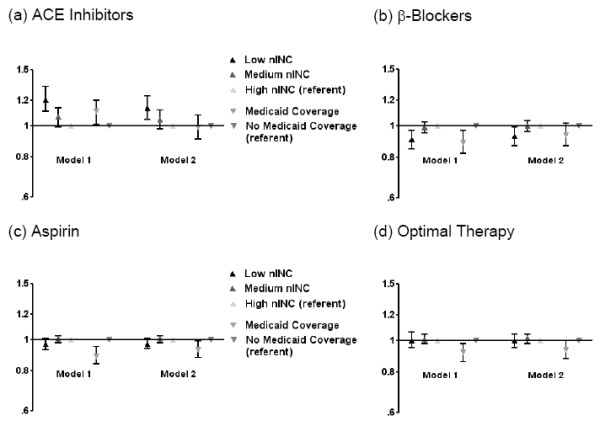
**Receipt of selected therapies among ARIC community surveillance patients (1993-2002)**. (a) ACE inhibitors. (b) β-blockers. (c) Aspirin. (d) Optimal therapy. Model 1: nINC, Medicaid status, race, gender, age, study community, year of MI. Model 2: Model 1 plus hospital type (teaching vs. non-teaching), current or past history of hypertension, diabetes or heart failure, and presence of cardiac pain

In models adjusted for race, gender, age, study community and year of MI, there was no significant effect modification of the nINC/Medicaid-therapy relationship by race, gender, age, study community or year of MI (p < 0.05). Low nINC was associated with a lower likelihood of being prescribed β-blockers at discharge (0.91, 0.86-0.97), but a higher likelihood of receipt of ACE inhibitors (1.20, 1.10-1.30) compared to high nINC. Similarly, patients with Medicaid coverage were more likely to receive ACE inhibitors (1.09, 1.00-1.18), but less likely to receive β-blockers (0.90, 0.82-0.97), aspirin (0.89, 0.84-0.94) or optimal therapy (0.92, 0.86-0.98) compared to patients without Medicaid coverage. Meanwhile, the likelihood of receipt of aspirin and optimal therapy among low nINC compared to high nINC patients did not reach statistical significance (Figure 1).

In models further adjusted for hospital type (teaching vs. non-teaching), current or past history of hypertension, diabetes or heart failure, and presence of cardiac pain with MI, associations between nINC and receipt of MI treatments were attenuated among low nINC (vs. high nINC) and patients as well as patients with Medicaid coverage (Figure 1). Low nINC remained associated with a lower likelihood of being prescribed β-blockers at discharge (0.93, 0.87-0.98), and a higher likelihood of receipt of ACE inhibitors (1.13, 1.04-1.22) compared to high nINC. Patients with Medicaid coverage were still less likely to receive aspirin (0.92, 0.87-0.98) and, with borderline significance, optimal therapy (0.94, 0.88-1.00), compared to patients without Medicaid coverage. Results for medium nINC compared to high nINC patients were not statistically significant (p < 0.05) in Model 1 or Model 2 (Figure 1). In fully adjusted models, study community, year of event, presence of cardiac pain and current or past history of heart failure were statistically significant predictors of receipt of both individual and optimal therapies.

## Discussion

Prior to this work, the association between SES and the receipt of pharmacologic therapy post-MI had not been examined via surveillance of CHD hospitalizations in the U.S. This analysis used both nINC and receipt of Medicaid to represent SES. In ARIC community surveillance, approximately 70% of Medicaid recipients live in low SES areas, as defined by census tract median household income[35,39]. However, in this analysis, nINC and Medicaid coverage had independent effects on the likelihood of receipt of evidence-based treatment post-MI. Despite a higher level of comorbidity, patients living in low nINC areas were less likely to be prescribed β-blockers at discharge compared to those living in high nINC census tracts, and patients with Medicaid coverage were less likely to be prescribed aspirin and optimal therapy compared to patients without Medicaid coverage.

Programs such as AHA's Get With the Guidelines (GWTG) are designed to improve the care of patients with cardiovascular and cerebrovascular diseases. Prior to the implementation of GWTG in 1,800 U.S. hospitals (1994-1995), patients' aspirin, ACE inhibitor and β-blocker use at discharge was 77.8%, 59.3% and 49.5%, respectively[40,41]. During a comparable time period, in our study, corresponding receipt rates were 87.6%, 36.6% and 60.9%.

A study by Rao and colleagues found that among Medicare beneficiaries, higher neighborhood income was correlated with higher rates of evidence-based medical treatment[42]. Conversely, a Canadian study found that access to cardiovascular medications among MI patients did not differ by neighborhood SES areas[43]. In contrast, our study captured the hospitalized MI experience of patients of varying ages with different levels of insurance coverage from communities across the U.S. We found higher neighborhood income was associated with an increased likelihood of being prescribed β-blockers during hospitalization among MI patients, and patients without Medicaid coverage were more likely to receive aspirin and optimal therapy. Thus, it is possible that nINC and Medicaid status operate independently via mechanisms not measured in this study, such as: patient self-efficacy and doctor-patient relationships, to influence the likelihood of receiving evidence-based treatment during hospitalization.

Our study employs data from ARIC community surveillance, the only ongoing population-based study in the U.S. which includes men and women representing a broad age range from biracial communities (Jackson, MS and Forsyth County, NC). The community-based surveillance design with systematic hospitalized MI event ascertainment minimizes selection bias for the current study. Limitations of data collected via hospital record abstraction include a lack of individual SES information, the use of Medicaid status as a proxy for individual SES, as well as an inability to adequately address the issue of contraindications for selected therapies. Further, the current study reflects the experience of in-hospital, not outpatient, treatment of MI in the ARIC surveillance communities.

It was surprising that the prescription of ACE inhibitors during the hospitalization or at discharge was higher for patients from low nINC areas compared to high nINC areas, especially considering the higher cost of ACE inhibitors compared to β-blockers at the time these data were collected[44] and the publication of research indicating that ACE inhibitors were less effective for lowering blood pressure in black compared to white patients[45]. These results are not consistent with a Swedish study in which high income patients were more likely to fill a prescription for ACE inhibitor therapy following a MI compared to low income patients[46]. However, our results are consistent with those reported by Rao et al in a comparison of low- and high-income patients[42].

In our study population, black patients represent the majority of patients living in low nINC areas. In addition, black patients had a high burden of comorbidities such as heart failure (40%) and hypertension (83%). Some researchers suggest a high risk of heart failure results in less frequent use of β-blockers and more frequent prescription of ACE inhibitors[47]. Although we adjusted for race, comorbidities and other sociodemographic and clinical factors, disparities in receipt of evidence-based therapies remained. It should be noted that we were not able to make inferences from ARIC data regarding the effect of financial incentives from pharmaceutical companies, and there is evidence that these and other contemporary issues may also influence physicians' prescribing patterns[48-51].

## Conclusions

In the current study, post-MI patients from socioeconomically disadvantaged communities and Medicaid recipients tended to receive individual and optimal treatments less often than patients from more affluent neighborhoods and non-Medicaid recipients. nINC and Medicaid coverage may be two of several socioeconomic factors influencing the complexities of medical care practice patterns.

## Abbreviations

ACC: American College of Cardiology; ACE: angiotensin converting enzyme; AHA: American Heart Association; ARIC: Atherosclerosis Risk in Communities; β: beta; CHD: coronary heart disease; GWTG: Get With the Guidelines; MD: Maryland; MI: myocardial infarction; MN: Minnesota; MS: Mississippi; NC: North Carolina; nINC: neighborhood median household income; PR: prevalence ratio; SES: socioeconomic status; U.S.: United States; 95% CI: 95% confidence interval.

## Competing interests

The authors declare that they have no competing interests.

## Authors' contributions

RF conceived of the study, performed the statistical analysis and drafted the manuscript. KR participated in the design of the study and critically revised the manuscript for important intellectual content. EW revised the manuscript for important intellectual content. CS participated in the design of the study and the interpretation of data. JW assisted in acquiring the data. WR participated in the design of the study and critically revised the manuscript for important intellectual content. All authors read and approved the final manuscript.

## Pre-publication history

The pre-publication history for this paper can be accessed here:

http://www.biomedcentral.com/1471-2458/10/632/prepub

## Supplementary Material

Additional file 1 **Supplemental Table S1. Characteristics of the Eligible Population by ARIC Study Community, 2000 Census**. A table of the study population and censual characteristics (total population, number of census tracts and average number of persons per census tract) of each ARIC study community. Click here for file
