# Supplementary material for: Neighborhood socioeconomic status, Medicaid coverage and medical management of myocardial infarction: Atherosclerosis risk in communities (ARIC) community surveillance
Source: BMC Public Health. 2010 Oct 21;10:632. doi: 10.1186/1471-2458-10-632 (PMC3201018; doi:10.1186/1471-2458-10-632)
Supplement: Additional file 1 — Supplemental Table S1. Characteristics of the Eligible Population by ARIC Study Community, 2000 Census. A table of the study population and censual characteristics (total population, number of census tracts and average number of persons per census tract) of each ARIC study community. [file 1471-2458-10-632-S1.doc]

Table S1. Characteristics (N) of the Eligible Population by ARIC Study Community, 2000 Census

|  | Washington Co. Maryland | Minneapolis  Minnesota | Jackson (city)  Mississippi | Forsyth Co.  North Carolina |
| --- | --- | --- | --- | --- |
|  |
| Race-gender composition |  |  |  |  |
| Black Women | 1,330 | 4,694 | 26,976 | 18,181 |
| Black Men | 1,220 | 4,380 | 21,545 | 15,175 |
| White Women | 29,048 | 48,329 | 8,491 | 53,272 |
| White Men | 27,033 | 45,168 | 7,137 | 47,887 |
| Total population^1^ | 58,631 | 102,571 | 64,149 | 134,515 |
|  |  |  |  |  |
| Number of census tracts | 31 | 55 | 43 | 75 |
| Average persons per census tract^1^ | 1,891 | 1,865 | 1,492 | 1,794 |
| Median Household Income^2^  Community-specific tertiles  High nINC  Medium nINC  Low nINC | $44,307  $46,761  $34,018-46,761  <$34,018 | $54,508  >$60,383  $50,032-60,383  <$50,032 | $25,480  >$30,727  $20,521-30,727  <$20,521 | $41,579  >$48560  $33,750-48,560  <$33,750 |

^1^ Limited to white and black persons 35 to 74 years of age.
^2^Calculated by averaging the median household income for each census tract in the area.
